# Unmet and unperceived needs for type 2 diabetes self-management among slum dwellers in Iran: a cross-sectional study

**DOI:** 10.1017/S1463423624000045

**Published:** 2024-03-14

**Authors:** Fawzieh Ghammari, Habib Jalilian, Masumeh Gholizadeh

**Affiliations:** 1 Department of Health Policy and Management School of Management and medical informatics, Tabriz University of medical sciences, Tabriz, Iran; 2 Department of Health Services Management, School of Health, Ahvaz Jundishapur University of Medical Sciences, Ahvaz, Iran; 3 Department of Health Policy and Management, School of Management and Medical Informatics, Tabriz University of Medical Sciences, Tabriz, Iran

**Keywords:** diabetes mellitus, diabetes self-management, disadvantaged population, slums, type 2 diabetes, unmet needs

## Abstract

**Aim:**

This study aimed to identify unmet and unperceived needs for T2D self-management among those residing in Tabriz slums, Iran, in 2022.

**Background:**

Type 2 diabetes (T2D) and its complications are more common among slum dwellers. T2D is a lifelong disease that requires continuous care. By contrast, slum dwellers are less likely to adhere to standard health care.

**Methods:**

This study is cross-sectional. We included 400 patients using a systematic random sampling method. Unmet and unperceived needs were assessed through a researcher-made questionnaire. The questionnaire was developed based on Iran’s Package of Essential Non-Communicable Diseases (IraPEN) instructions and an expert panel. Data were analyzed using SPSS version 22.

**Findings:**

Need for more healthcare cost coverage by insurance organizations (85.5%), financial support to provide medicine (68%), free and accessible sports equipment in the area (48.5%), continuous access to blood sugar test instruments (47.8%), know how to test blood sugar and interpret the results (47.7%), more communication with healthcare providers (42.3%), and detailed education from health professionals (41.2%) were the most common unmet needs. The least perceived need was to know how to care for feet (16%).

## Introduction

Type 2 diabetes (T2D) is one of the world’s most common non-communicable diseases (NCDs) (Ahmad *et al*., 2022). T2D accounts for 12% of world health expenditure and is the fifth death cause in the world (International Diabetes Federation, 2015). T2D is a lifelong disease that affected over 425 million worldwide in 2017 and is expected to rise to 629 million people by 2045 (Zhang and Gregg, 2017). More than 75% of those with T2D live in developing countries (World Health Organization, 2007).

T2D, its risk factors, and complications are more common among disadvantaged groups (IDF Diabetes Atlas, 2017). People with low socioeconomic status (SES), those belonging to ethnic minorities, and those with low health literacy and insufficient skills in disease management can be considered disadvantaged populations (National Academies of Sciences and Medicine, 2015). Slum dwellers are disadvantaged populations who are characterized by low income, poor housing conditions, overcrowding, poor health outcomes, and inadequate access to health and basic facilities (UN-Habitat, 2016). Due to these characteristics, slums have been considered a global health priority in the United Nations’ Sustainable Development Goals (UN SDGs) (Improving Health in Slums Collaborative, 2021).

Slum dwellers are exposed to some conditions and behaviors which increase the chance of being afflicted by T2D. In other words, social and environmental factors might influence access to and acceptance of healthy behaviors (Chan *et al*., 2022). A study in Kenya demonstrated the weighted prevalence of behavioral factors contributing to NCD expansion among slum dwellers, including unhealthy diet (57.2%), low exercise (14.4%), alcohol abuse (10.1%), and tobacco use (12.4%) (Haregu *et al*., 2015). Results of a study in Brazil showed that the prevalence of T2D among slum dwellers is nearly two-fold compared to the rest of the population (10.1% vs. 5.2%) (Snyder *et al*., 2017). Despite the high level of healthcare needs among those with T2D living in slums, they are less likely to adhere to standard health care (Seghieri *et al*., 2019).

T2D self-management is complicated and needs multifaceted actions. It includes regular care and activities such as physical activity, adherence to prescribed treatment, and monitoring glucose levels (Shin and Lee, 2018). The role of patients in optimal diabetes self-management is focal. Due to the socioeconomic characteristics of slum dwellers and less adherence to appropriate care, identifying unperceived and unmet needs for proper planning in slums is necessary. Achievement of this goal can be helpful for optimal diabetes self-management and prevention of diabetes complications.

Tabriz is a metropolis in Iran, and the number of its slum dwellers has recently increased. A study in Tabriz showed that because of high costs, underutilization of health services is more common among slum dwellers compared to the general population (7.2% vs. 3.3%) (Tabrizi *et al*., 2018). Also, the results of a study in Tabriz showed that the underutilization of health services among slum dwellers with T2D is considerable (Ghammari *et al*., 2023b).

Given the special characteristics of slum dwellers and their special needs, this study aimed to identify unmet and unperceived needs for T2D self-management among them in Tabriz, Iran, in 2022.

## Materials and methods

### Study design and setting

This study is cross-sectional that was conducted among those with T2D living in Tabriz slums in 2022. Patients were included if they had over 18 years old and lived for over 5 years in urban slums. Those with other types of diabetes and those with psychological disabilities were excluded.

Tabriz with over 2 million people is one of Iran’s most populous cities. According to the Vice-Chancellor for Health of Tabriz University of Medical Sciences, over 380 thousand people lived at the time of the study in Tabriz slums, of which 13 155 were diabetic.

### Sample size and sampling

374 patients were needed to conduct the study, according to Cochran’s Sample Size Formula (Cochran, 1977) with a 95% confidence level and 0.05 marginal error. A list of those with T2D was extracted from an integrated health system, called the SIB, and they were assessed for inclusion criteria. Iran’s Ministry of Health established the SIB to record, maintain, and update electronic health records for Iranians. The SIB allows doctors and health experts to manage patients’ diseases and facilitate referrals. Patients’ primary information such as age, gender, phone number, and address are recorded at SIB. In the Tabriz slums, there are four health complexes. Nearly 100 patients were selected from each health complex using a systematic random sampling method. At last, 400 people were the final participants in the study. Two researchers interviewed patients in their homes for 2 months.

### Data collection tools and data analysis

For data collection, a questionnaire was designed. The potential needs were identified based on Iran’s Package of Essential Non-Communicable Diseases (IraPEN) instructions and an expert panel including 3 members of the Iranian diabetes society, 4 general practitioners, 5 primary healthcare providers, and 6 representatives of slum-dweller diabetic patients. IraPEN is a plan for regular care and prevention of NCDs that was launched by Iran’s Ministry of Health in 2014. IraPEN has suggested essential care needs for diabetic patients. Finally, 25 needs were included in the questionnaire. These needs were categorized into four principal categories: educational, financial, social, and access. Cronbach’s alpha for the questionnaire reliability was 0.872. The face validity of the questionnaire was confirmed by experts in the panel. The questionnaire consisted of two parts. The first part included sociodemographic questions (11 items). Perceived needs and met needs were asked in the second part. Perceived needs refer to services that patients think are necessary for optimal diabetes self-management. Met needs refer to services received. Unmet needs are the differences between met needs and perceived needs. Literate people completed the questionnaire themselves, but for illiterate ones, the questions were read by two researchers. Data analysis was conducted using SPSS version 22. Descriptive statistics (frequency and percent) were used to assess the extent of unmet and perceived needs. We examined the associations between sociodemographics and perceived and unmet needs using an independent two-sample t-test for variables with two categories and one-way ANOVA for variables with more than two categories. To this end, we compiled the questions into four categories by assigning a zero score to perceived needs and a one score to unperceived needs. After that, the sum of the scores of the questions in each category was divided by the number of questions in that category. Also, unmet needs were categorized into four categories, with a score of zero assigned to unmet needs and a score of one for met needs. In each category, the sum of the questions’ scores was divided by the number of questions.

## Results

### Sociodemographic characteristics

More than half of 400 patients were female and over 90% were over 30 years. Over 58% of patients were illiterate or were able to read and write. More than 75% had income below 40 million rials and 85% weren’t covered by supplemental insurance (Table 1).

**Table 1.** Sociodemographic characteristics of the study population

Variables	Categories	Frequency (percent)
Gender	Male	185 (46.3)
	Female	215 (53.8)
Age (in years)	Under 30	38 (9.5)
	30–40	91 (22.8)
	40–50	82 (20.5)
	50–60	87 (21.8)
	Over 60	102 (25.5)
Education	Illiterate	111 (27.8)
	Reading and writing ability	121 (30.3)
	Diploma	131 (32.8)
	Academic education	37 (9.3)
Marital status	Single	40 (10.0)
	Married	308 (90.0)
Income (The Iranian Rial)	<40 million	303 (75.8)
	40–80 million	78 (24.3)
Basic Insurance	Yes	329 (82.3)
	No	71 (17.8)
Type of Insurance	Social Security Insurance	193 (57.8)
	Iran health insurance	114 (34.1)
	Others	27 (8.1)
Supplemental Insurance	Yes	60 (15.0)
	No	340 (85.0)
Disease duration (years)	>5	114 (28.5)
	5–9	104 (26.0)
	10–14	55 (13.8)
	15–20	40 (10.0)
	<20	46 (11.5)
Treatment type	Lifestyle change	27 (6.8)
	Oral pills	236 (59.0)
	Insulin	17 (4.3)
	Mixed regime simultaneously	120 (30.0)
Diabetes complications	Yes	286 (71.5)
	No	114 (28.5)

### Unmet and unperceived needs

There are nine needs in the educational category. The most considerable unmet needs were the need to know how to test blood sugar and interpret the results (47.7%), the need for detailed education from health professionals (41.2%), and the need for know how to measure blood pressure (31%). The least frequently unmet need is on how to care foot (−12.5%), which is perceived at the least.

The financial category consists of 4 needs. The need for more healthcare cost coverage by insurance organizations (85.5%), the need for financial support to provide medicine (68%), and the need for financial support to provide healthy foods (30.5%) were the most critical unmet needs.

There are five needs in the social category. More communication with healthcare providers (42.3%) and a safe area for physical activity (24.2%) are the most common unmet needs.

The access needs consist of 7 needs. The need for free and accessible sports equipment in the area (48.5%), the need for continuous access to blood sugar test instruments (47.8%), and the need for continuous access to diagnostic and treatment services (33.8%) had the highest frequency (Table 2).

**Table 2.** Unmet and unperceived needs for T2D self-management among slum dwellers in Tabriz

Needs	Perceived needs		Met needs		Unmet needs (percent)
	Frequency	Percent	Frequency	Percent	
**Educational needs**					
Need for information about T2D and its complications	336	91.5	274	68.5	23
Need for information about the importance of treatment adherence and lifestyle changes	356	86.5	271	67.8	18.7
Need for information about healthy diet	360	90	296	74	16
Need for information about the importance of the physical activity	326	81.5	284	71	11.5
Need for know how to test blood sugar and interpret the results	312	78	121	30.3	47.7
Need for know how to measure blood pressure	208	52	82	20.5	31.5
Need for know how to take medicine and insulin	275	68.8	280	70	−1.2
Need for detailed education from health professionals	381	95.3	218	54.5	41.2
Need for know how to care foot	64	16	114	28.5	−12.5
**Financial needs**					
Need for financial support to provide medicine	338	84.5	66	16.5	68
Need for financial support to provide healthy foods	254	63.5	120	30	30.5
Need for financial support to provide insulin	104	26	7	1.8	24.2
Need for more healthcare cost coverage by insurance organizations	375	93.8	33	8.3	85.5
**Social needs**					
Need for a safe area for physical activity	316	79	225	56.3	24.2
Need for social support from family and friends	381	95.5	324	81	14.5
Need for psychological support from family and friends	384	96	324	81	15
Need for peer group	182	45.5	104	26	19.5
Need for more communication with healthcare providers	337	84.3	168	42	42.3
**Access needs**					
Need for free and accessible sports equipment in the area	322	80.5	128	32	48.5
Need for Continuous access to doctors in the area	354	88.5	252	63	25.5
Need for continuous follow-up from health centers	211	52.8	150	37.5	15.3
Need for continuous access to diagnostic and treatment services	353	88.3	218	54.5	33.8
Need for access to a psychologist	128	32	92	23	9
Need for continuous access to diabetes expert	266	66.5	171	42.8	23.7
Need for continuous access to blood sugar test instruments	323	80.8	132	33	47.8

As shown in Table 3, most perceived needs are social (Mean = 0.79, SD = 0.23), and most unmet needs are financial (Mean = 0.85, SD = 0.23). Education has the lowest perceived need (Mean = 0.65, SD = 0.19), while social needs have the lowest unmet need (Mean = 0.44, SD = 0.28).

**Table 3.** Means of perceived and unmet needs for T2D self-management among slum dwellers in Tabriz

Needs	Perceived	Unmet
	Mean ± SD	Mean ± SD
Educational needs	0.65 ± 0.19	0.49 ± 0.25
Financial needs	0.66 ± 0.24	0.85 ± 0.23
Social needs	0.79 ± 0.23	0.44 ± 0.28
Access needs	0.68 ± 0.20	0.60 ± 0.27

### Association between sociodemographic characteristic and perceived and unmet needs

Perceived educational needs are associated with low income, lack of basic insurance, and lack of supplemental insurance. Perceived financial needs are associated with higher age, income, and lack of basic and supplemental insurance. Perceived social needs are associated with women’s gender, older age, being single, lower income, lack of basic and supplemental insurance, and coverage by Iran Health Insurance. Perceived access needs were associated with older age, lower income, lack of basic and supplemental insurance, and coverage by Iranian Health Insurance (Table 4).

**Table 4.** Association between sociodemographic characteristics and perceived needs

Variables	categories	Educational		Financial		Social		Access	
		Mean ± SD	*P*-value	Mean ± SD	*P*-value	Mean ± SD	*P*-value	Mean ± SD	*P*-value
Gender	Male	0.49 ± 0.26	0.72	0.86 ± 0.23	0.78	0.39 ± 0.26	0.001^a^	0.59 ± 0.27	0.48
	Female	0.49 ± 0.24		0.85 ± 0.23		0.49 ± 0.29		0.61 ± 0.27	
Age	>30	0.50 ± 0.16	0.74	0.83 ± 0.22	0.001^a^	0.30 ± 0.23	<0.0001^a^	0.56 ± 0.24	0.002^a^
	30-60	0.48 ± 0.25		0.83 ± 0.24		0.42 ± 0.27		0.58 ± 0.27	
	<60	0.50 ± 0.28		0.93 ± 0.17		0.56 ± 0.27		0.69 ± 0.26	
Marital Status	Single	0.50 ± 0.26	0.76	0.83 ± 0.24	0.37	0.50 ± 0.31	0.02^a^	0.60 ± 0.29	0.85
	Married	0.49 ± 0.25		0.86 ± 0.22		0.43 ± 0.27		0.61 ± 0.26	
Income	<40 million	0.52 ± 0.25	<0.0001^a^	0.88 ± 0.21	<0.0001^a^	0.49 ± 0.27	<0.0001^a^	0.65 ± 0.26	<0.0001^a^
	40-80 million	0.37 ± 0.21		0.88 ± 0.27		0.28 ± 0.23		0.46 ± 0.24	
Basic insurance	Yes	0.45 ± 0.24	<0.0001^a^	0.83 ± 0.24	<0.0001^a^	0.40 ± 0.27	<0.0001^a^	0.56 ± 0.26	<0.0001^a^
	No	0.68 ± 0.20		0.95 ± 0.11		0.64 ± 0.24		0.80 ± 0.21	
Supplemental insurance	Yes	0.35 ± 0.22	<0.0001^a^	0.77 ± 0.25	0.002^a^	0.27 ± 0.24	<0.0001^a^	0.44 ± 0.23	<0.0001^a^
	No	0.52 ± 0.25		0.87 ± 0.22		0.47 ± 0.27		0.63 ± 0.27	
Type of insurance	Social security insurance	0.43 ± 0.22	0.07	0.84 ± 0.23	0.93	0.38 ± 0.26	0.02^a^	0.53 ± 0.25	0.01^a^
	Iran health insurance	0.49 ± 0.26		0.83 ± 0.25		0.41 ± 0.26		0.62 ± 0.28	
	Others	0.42 ± 0.25		0.82 ± 0.26		0.53 ± 0.32		0.53 ± 0.27	

a Significant was considered for *P*-value <0.05

Unmet educational needs are associated with higher age and lack of basic insurance. Unmet financial needs are associated with higher age, lower income, lack of basic and supplemental insurance, and coverage by Iran Health Insurance. Unmet social needs are associated with older lower income, and lack of basic insurance. There was an association between unmet access needs and the gender of women (Table 5).

**Table 5.** Association between sociodemographic characteristics and unmet needs

Variables	Categories	Educational		Financial		Social		Access	
		Mean ± SD	*P*-value	Mean ± SD	*P*-value	Mean ± SD	*P*-value	Mean ± SD	*P*-value
Gender	Male	0.65 ± 0.21	0.77	0.65 ± 0.25	0.13	0.78 ± 0.28	0.38	0.65 ± 0.22	0.02^a^
	Female	0.65 ± 0.18		0.68 ± 0.22		0.80 ± 0.22		0.70 ± 0.18	
Age	>30	0.56 ± 0.11	0.007^a^	0.59 ± 0.26	0.001^a^	0.71 ± 0.26	<0.0001^a^	0.69 ± 0.14	0.83
	30-60	0.65 ± 0.19		0.65 ± 0.23		0.80 ± 0.22		0.67 ± 0.21	
	<60	0.67 ± 0.23		0.74 ± 0.22		0.90 ± 0.12		0.67 ± 0.21	
Marital status	Single	0.61 ± 0.20	0.057	0.67 ± 0.27	0.94	0.76 ± 0.26	0.28	0.68 ± 0.21	0.72
	Married	0.66 ± 0.19		0.66 ± 0.23		0.79 ± 0.22		0.67 ± 0.20	
Income	<40 million	0.64 ± 0.20	0.14	0.72 ± 0.21	<0.0001^a^	0.89 ± 0.18	<0.0001^a^	0.67 ± 0.21	0.26
	40-80 million	0.67 ± 0.17		0.51 ± 0.25		0.75 ± 0.23		0.70 ± 0.17	
Basic insurance	Yes	0.59 ± 0.24	0.01^a^	0.64 ± 0.25	<0.0001^a^	0.72 ± 0.25	0.004^a^	0.68 ± 0.20	0.26
	No	0.66 ± 0.18		0.76 ± 0.13		0.80 ± 0.22		0.65 ± 0.23	
Supplemental insurance	Yes	0.67 ± 0.17	0.36	0.46 ± 0.27	<0.0001^a^	0.83 ± 0.22	0.17	0.68 ± 0.15	0.85
	No	0.64 ± 0.20		0.70 ± 0.21		0.78 ± 0.23		0.67 ± 0.21	
Type of insurance	Social security insurance	0.66 ± 0.17	0.90	0.61 ± 0.27	0.006^a^	0.82 ± 0.22	0.14	0.69 ± 0.19	0.61
	Iran health insurance	0.66 ± 0.18		0.70 ± 0.20		0.77 ± 0.21		0.66 ± 0.21	
	Others	0.67 ± 0.24		0.68 ± 0.23		0.80 ± 0.21		0.69 ± 0.24	

a Significant was considered for *P*-value <0.05.

## Discussion

This study aimed to identify unmet and unperceived needs for T2D self-management among slum dwellers. The results of the study show the importance of financial, educational, social, and access factors for T2D self-management among slum dwellers.

Sociodemographic characteristics of the study population remind us of slum dwellers, known as Tom-all-alone in Dickens’s novel (Dickens, 1853). Most of them have poor education and are afflicted by diabetes complications. All of the participants in the study are below the poverty line defined by Iran. These characteristics often affect T2D self-management and should be considered as unmet needs for T2D self-management in slums.

The findings indicated that perceived educational, financial, social, and access needs, as well as unmet financial and social needs are significantly associated with low income. In light of this, it emphasizes the importance of providing financial support to slum dwellers with T2D. Our study showed a noticeable difference between perceived and met needs for more healthcare cost coverage by insurance organizations (85.5%). Poor health insurance coverage, high co-payments, and distrust in the healthcare system are barriers to appropriate diabetes self-management in disadvantaged groups (Williams, 2011; Vest *et al*., 2013). Additionally, the findings indicate that unmet financial, social, and access needs are associated with a lack of basic and supplemental insurance and Iran Health Insurance coverage. There is no adequate coverage of costs nor a sufficient benefits package in Iran Health Insurance. It is therefore necessary to provide basic insurance to slum dwellers and provide support until they are able to purchase supplemental insurance. In addition, for appropriate diabetes self-management, more cost coverage special for slum dwellers diabetics is necessary. In addition, the study results showed that those living in urban slums face financial constraints to provide medicine, healthy diets, and insulin. One of the most critical behaviors for successful disease management and preventing diabetes complications is adhering to a treatment regime (Javanmardifard *et al*., 2020) including regularly taking medicine. A primary barrier to adhering to the treatment regime is the high cost of medicines (Vest *et al*., 2013).

Although one of IraPEN’s goals is to provide regular care and education for diabetic patients (World Health Organization, 2020), these needs remain considerably unmet among slum dwellers. This should be interpreted with caution. The results of a systematic review showed that T2D self-management was impacted by the individual, health system, and contextual factors (Ghammari *et al*., 2023a). Maybe a unilateral plan from the government does not guarantee meeting goals and a plan that involves the health system, patients, and families is necessary.

Adopting an appropriate lifestyle including a healthy diet is a focal action for diabetes self-management. The American Diabetes Association (ADA) has recommended that an appropriate diet for adults with T2D should focus on healthy diet patterns based on nutrition principle to achieve a healthy weight, appropriate levels of glycosylated hemoglobin (HbA1c), normal blood pressure, and normal lipid index (American Diabetes Association, 2018). To this end, patients should know the healthy foods and their impacts on diabetes. Along with financial factors, education is an important aspect of diabetes self-management improvement in urban slums.

Although many patients have broadly perceived the need for education on how to test blood sugar correctly and interpret the results, most have known it is unmet. Therefore, the importance of education appears. A study showed that short-course education positively affects glycemic control (Afshar and Mirbagher-Ajorpaz, 2012). The study showed the importance of detailed education from health providers. Several studies have demonstrated the importance of education on diabetes and its complications, a healthy diet, and regular treatment for optimal diabetes self-management in urban slums. To this end, should consider educational material tailored to people’s literacy levels, and community-based learning (Rose *et al*., 2004; Gazmararian *et al*., 2009; Tiedt and Sloan, 2015). Our study disclosed that although foot care in diabetic patients is critical to prevent adverse complications, this need is less perceived among slum dwellers (16%). Patients in Mumbai slums reported that a lack of information regarding foot care is a barrier to self-care practices (Shah *et al*., 2021). The prevalence of diabetic foot ulcer is one of the most common diabetes complications with 4 to 10% (Solan *et al*., 2017). Diabetes foot ulcer accounts for 40% of the total estimated cost of diabetes in developing countries (Jeffcoate *et al*., 2018). Therefore, attention to unperceived needs that affect optimal diabetes self-management is necessary. Regular awareness about the importance of daily foot care and education on how to care foot is recommended.

The role of social factors in diabetes self-management is clear. The study shows that patients broadly perceived the need for social and psychological support from family and friends (96%). Results of a study in Bulgaria showed that 50% of those with T2D need higher psychological support from family for better diabetes self-management (Yordanova *et al*., 2015). Results of a study in Vietnam showed that the odds of diabetes-related distress are higher among those with T2D who have had an unmet need for social support (Thi *et al*., 2021). Support from family and friends is a facilitator for diabetes self-management and better treatment adherence (Adisa *et al*., 2017; Baghikar *et al*., 2019; Mphwanthe *et al*., 2021). Family support positively affects nutrition, psychological conditions, control of blood sugar, diabetes management, and health outcomes among those with uncontrolled glycemic (Pamungkas *et al*., 2017). Study results showed an association between being female and older and unmet and perceived social needs. As a result of these findings, more attention needs to be paid to women and older people when it comes to appropriate self-management of diabetes.

The importance of communication between patients and providers is a perceived need that is unmet. Other studies showed better relationships could improve diabetes self-management among slum dwellers (Bhojani *et al*., 2013; Jackson *et al*., 2017; Ghammari *et al*., 2023c).

Although diabetes is psychologically challenging (Perrin *et al*., 2017), the need for access to a psychologist was only perceived by 32% of slum dwellers. Therefore, slum dwellers notably need to have consistent education and develop skills in diabetes self-management.

One of the actions for optimal diabetes self-management is regular physical activity (Sigal *et al*., 2004). Slum dwellers broadly perceived the importance of a safe physical activity environment but found neighborhoods unsafe. Safe areas can be an affecting factor in improving exercise in disadvantaged populations (Power *et al*., 2020; Leyns *et al*., 2021). The need for peer groups is perceived by 45.5% of slum dwellers. They can share their information and experiences about T2D. In addition, they can benefit from each other financial support. Given low SES among slum dwellers, creating and strengthening peer groups is necessary (Masupe *et al*., 2018; Mphwanthe *et al*., 2021). To this end, the use of slum capacities such as mosques and leaders can be helpful.

According to the results, access to health services and sports facilities to improve diabetes self-management is necessary. In urban slums, diabetes self-management is negatively impacted by the lack of access to sports facilities and healthcare services (Kotian *et al*., 2019). Therefore, these matters need special attention in urban slums. A big part of these issues is beyond the sphere of health systems.

## Strengths and limitations

The study shows a comprehensive picture of unmet needs for diabetes self-management among those living in urban slums. Because of the socioeconomic status similarity between urban slums, the results can be generalized and used in other urban slums. There is one major limitation to the study, which was conducted only among patients.

## Conclusions

Our results showed that some important needs for diabetes self-management have not been met or perceived among slum dwellers. To improve diabetes self-management, it is important to consider unmet and unperceived needs in the financial, educational, social, and access categories. Collaboration between the health system, government, and insurance organizations for developing a comprehensive plan is necessary to change the status quo. Detailed and continuous education about diabetes self-management and required activities/behaviors for maintaining a healthy life should be provided to patients and their families. Insurance companies should increase coverage of costs for those with T2D living in urban slums. To provide medicine, healthy foods, and insulin to slum dwellers, the government and health system should provide special subsidies. To this end, they can utilize innovative financing and national and international aid. Finally, the government should consider upgrading urban slums for more access to healthcare and sports facilities. At last, we recommend a qualitative study among slum dwellers for a deeper understanding of enablers and barriers to diabetes self-management.
